# H^+^ channels in embryonic *Biomphalaria glabrata* cell membranes: Putative roles in snail host-schistosome interactions

**DOI:** 10.1371/journal.pntd.0005467

**Published:** 2017-03-20

**Authors:** Brandon J. Wright, Utibe Bickham-Wright, Timothy P. Yoshino, Meyer B. Jackson

**Affiliations:** 1 Department of Neuroscience, School of Medicine and Public Health, University of Wisconsin-Madison, Madison, Wisconsin, United States of America; 2 Department of Pathobiological Sciences, School of Veterinary Medicine, University of Wisconsin-Madison, Madison, Wisconsin, United States of America; George Washington University School of Medicine and Health Sciences, UNITED STATES

## Abstract

The human blood fluke *Schistosoma mansoni* causes intestinal schistosomiasis, a widespread neglected tropical disease. Infection of freshwater snails *Biomphalaria spp*. is an essential step in the transmission of *S*. *mansoni* to humans, although the physiological interactions between the parasite and its obligate snail host that determine success or failure are still poorly understood. In the present study, the *B*. *glabrata* embryonic (Bge) cell line, a widely used *in vitro* model for hemocyte-like activity, was used to investigate membrane properties, and assess the impact of larval transformation proteins (LTP) on identified ion channels. Whole-cell patch clamp recordings from Bge cells demonstrated that a Zn^2+^-sensitive H^+^ channel serves as the dominant plasma membrane conductance. Moreover, treatment of Bge cells with Zn^2+^ significantly inhibited an otherwise robust production of reactive oxygen species (ROS), thus implicating H^+^ channels in the regulation of this immune function. A heat-sensitive component of LTP appears to target H^+^ channels, enhancing Bge cell H^+^ current over 2-fold. Both Bge cells and *B*. *glabrata* hemocytes express mRNA encoding a hydrogen voltage-gated channel 1 (HVCN1)-like protein, although its function in hemocytes remains to be determined. This study is the first to identify and characterize an H^+^ channel in non-neuronal cells of freshwater molluscs. Importantly, the involvement of these channels in ROS production and their modulation by LTP suggest that these channels may function in immune defense responses against larval *S*. *mansoni*.

## Author summary

*Schistosoma mansoni* is one of four major species of human blood flukes that, together, infect over 250 million people worldwide. Transmission of *S*. *mansoni* to humans requires infection of freshwater intermediate host snails, *Biomphalaria* spp., in order to complete its life cycle. The *B*. *glabrata* embryonic (Bge) cell line, derived from a Puerto Rican strain of snail host shares characteristics with circulating hemocytes, the molluscan immune cells, and serves as an *in vitro* model for snail immune function. Electrical recordings from Bge cells demonstrated the presence of H^+^ channels that allow hydrogen ions (H^+^) to cross the membrane. Furthermore, blocking these channels inhibited the production of reactive oxygen species (ROS), an immune defense mechanism shared by Bge cells and hemocytes. Interestingly, Bge cell exposure to proteins produced by *S*. *mansoni* larvae exerted the opposite effect, enhancing H^+^ movement across the cell membrane. An H^+^ channel-encoding gene was expressed in both Bge cells and hemocytes suggesting that hemocytes may share similar functions with Bge cells.

## Introduction

Schistosomiasis, a neglected tropical disease afflicting over 250 million people worldwide [1], is caused by parasitic flatworms of the genus *Schistosoma*. *Schistosoma* spp. have a two-host life cycle involving sexual reproduction within a mammalian host and asexual reproduction within a snail intermediate host. The pathology associated with the intestinal form of human schistosomiasis arises in chronic infections when eggs released by female worms occupying mesenteric veins become trapped in the liver (and other organs) and elicit an intense inflammatory response leading to the formation of granulomas that damage tissues and block circulation [2, 3]. Eggs from ruptured intestinal capillaries exit the host by fecal excretion, and upon exposure to freshwater, hatch to release the free-swimming snail-infective miracidia. Upon infection of snails, miracidia transform through two sporocyst stages, ultimately completing their life cycle by the production and release of free-swimming cercariae, the human-infective stage [4]. Because of the absolute dependency of human schistosome transmission on the snail host, one of the keys to sustained control of schistosomiasis is to block or eliminate the snail’s participation in the life cycle.

The freshwater snail *Biomphalaria glabrata* serves as the most common invertebrate host of *S*. *mansoni*, the most widely distributed species of *Schistosoma* [5]. Hemocytes (phagocytic immune cells) of *B*. *glabrata*, genetically-selected for susceptibility or resistance to infection by larval *S*. *mansoni*, have been shown to react differentially to invading miracidia. Circulating hemocytes of susceptible strains do not recognize and kill invading larvae, whereas in resistant snails developing larvae are rapidly encapsulated by hemocytes and killed within 24–48 hours of infection [6–8]. Hemocyte larvicidal activity has been linked to the production and release of reactive oxygen species (ROS), mainly hydrogen peroxide (H_2_O_2_), and the reactive nitrogen species, nitric oxide [9, 10]. Although hemocytes of both resistant and susceptible *B*. *glabrata* strains produce H_2_O_2_, resistant hemocytes generate and release higher levels than susceptible cells [11], and this production appears to depend on the extracellular signal regulated protein kinase (Erk) [12]. However, a critical question arising from these observations is what are the signaling mechanisms that regulate ROS responses?

A critical period of larval development in the snail host is 24–48 hours post-infection, when the newly invading miracidium completes its transformation to the primary sporocyst stage. Larval killing depends on the ability of circulating hemocytes to recognize and encapsulate the newly formed sporocyst [4, 13–15]. Among various sporocyst factors that may be contributing to hemocyte reactivity are glycoproteins that are released during the miracidium-to-sporocyst transition. *In vitro* studies have shown that these larval transformation proteins (LTPs) [16] modulate phagocytic activity, motility, and ROS production in *B*. *glabrata* hemocytes [17–21], and disrupt hemocyte immune signaling [22–24]. However, questions regarding specific mechanisms by which LTPs modulate hemocyte immune responses remain unanswered.

For over four decades a cell line derived from embryos of a schistosome-susceptible strain of *B*. *glabrata*, the *B*. *glabrata* embryonic (Bge) cell line [25], has served as an *in vitro* model for the study of larval schistosome-snail host interactions in schistosomiasis. Bge cells share many characteristics with *B*. *glabrata* hemocytes including their morphology, adhesive properties, phagocytic activity, and larval encapsulation response [26]. In fact, co-culture of Bge cells with *S*. *mansoni* larvae results in the development of the parasite from the miracidium to the final cercarial stage, similar to the development that occurs with susceptible *B*. *glabrata* strains [27–30]. We have therefore adopted Bge cells as an *in vitro* model system to study the molecular interactions between snail cells and *S*. *mansoni* LTP. Because ion channels in the plasma membrane of human immune cells, including eosinophils, macrophages, neutrophils and lymphocytes, play important roles in immune responses, often by regulating the production and release of ROS [31], we explored the role ion channels may play in signaling and ROS production in Bge cells. Using the whole cell patch clamp technique, we discovered an LTP-sensitive H^+^ channel that serves as the dominant ion conductance of Bge cell membranes. In addition, using a fluorescent probe to measure intracellular ROS, we also found that this channel mediates the production of ROS, thus suggesting a possible function for H^+^ channels in snail immune responses.

## Materials and methods

All animal care and procedures were approved by the Institutional Animal Care and Use Committee of the University of Wisconsin-Madison under protocol V00640.

### Maintenance of Bge cells

The Bge cell line was originally obtained from American Type Culture Collection (ATCC CRL 1494) and is currently available through the BEI Resources (https://www.beiresources.org). Cells were maintained at 26°C under normoxic conditions in complete Bge (c-Bge) medium consisting of 22% Schneider’s *Drosophila* Medium, 0.45% lactalbumin enzymatic hydrolysate, and 7.2 mM galactose supplemented with 10% heat-inactivated fetal bovine serum and 1% penicillin/streptomycin [25, 28]. Bge cells were passaged at 80% confluency.

### Collection of larval transformation proteins (LTPs)

*S*. *mansoni* eggs were isolated, hatched, and miracidia cultured *in vitro* as previously described [28]. Approximately ~ 5000 miracidia/mL in Chernin’s balanced salt solution (CBSS; 47.9 mM NaCl, 2.0 mM KCl, 0.5 mM Na_2_HPO_4_, 0.6 mM NaHCO_3_ 1.8 mM MgSO_4_, 3.6 mM CaCl_2_ and pH 7.2) [32] supplemented with glucose (1 mg/mL), trehalose (1 mg/mL), penicillin G (100 units/mL) and streptomycin sulfate (0.05 mg/mL) adjusted to pH 7.2 (CBSS+) were then plated in a 24-well tissue culture plate and incubated at 26°C under normal atmospheric conditions to allow *in vitro* transformation of miracidia to primary sporocysts. The LTP-containing culture medium was collected after 48 hr, and the newly transformed primary sporocysts were washed once with CBSS+. The LTP and CBSS+ wash were combined, filtered with a 0.45 μm Nalgene syringe filter (Thermo Scientific, Waltham, MA), and concentrated using 3 kDa molecular weight cut-off ultrafiltration tubes (Amicon Ultra Centricon, Billerica, MA). A NanoDrop ND-1000 spectrophotometer (NanoDrop Technologies, Wilmington, DE) was used to determine the protein concentration, after which a protease inhibitor cocktail (Calbiochem, Billerica, MA) was added. Multiple collections of LTP were pooled and stored in aliquots at -20°C. To denature LTP, pools were boiled at 100°C for 5 min.

### Patch-clamp recording

Bge cells (~4 x 10^6^) were plated in 60x15 mm petri dishes in c-Bge medium, and allowed to attach overnight. In order to make recordings under defined ionic conditions, cells were washed 3X with CBSS before recording and kept in this buffer during subsequent manipulations. In experiments involving the treatment of Bge cells with ZnCl_2_, 10 mM HEPES replaced NaH_2_PO_4_ in CBSS due to the insolubility of Zn_3_(PO_4_)_2_. Adherent cells were viewed with an Axioskop microscope equipped with a 63X water-immersion objective (Carl Zeiss, Thornwood, NY). Bge cells were imaged with a CCD camera and viewed on a monitor. Patch electrodes fabricated from borosilicate glass capillaries had resistances of 3–7 MΩ when filled with a solution containing (in mM) 60 K-gluconate, 1 CaCl_2_, 1 MgCl_2_, 1 Mg-ATP, 10 HEPES, and 5 EGTA. The bathing solution for recordings was a slightly modified version of CBSS consisting of (in mM): 47 NaCl, 2 KCl, 0.5 NaH_2_PO_4_, 0.6 NaHCO_3_, 1.8 MgSO_4_, 3.6 CaCl_2_. The pH of the pipette solution and external CBSS was adjusted to 5 or 7 with KOH or HCl. Modified versions of the internal and external solutions are stated in the Results section where they are used. Pressure-ejection pipettes were modified patch electrodes with tip diameters of ~2 μm. A Picospritzer II (General Valve Corp.) was used to apply 5–10 PSI of pressure to ejection pipettes.

Patch clamp recordings were made with an Axopatch 200B amplifier (Molecular Devices, Sunnyvale, CA), with data read into a PC through a Digidata 1440 A interface. The computer program pClamp 10 (Molecular Devices) controlled data acquisition, voltage steps, and pressure application by the Picospritzer. Data were filtered with a low-pass Bessel filter at 2 kHz before digitization at 10 kHz.

### Reactive oxygen species (ROS) measurement

The fluorescent probe 2’7’-dichlorofluorescein-diacetate (DCFH-DA; Sigma-Aldrich, St. Louis, MO) was used to measure ROS production in Bge cells following a method described previously with hemocytes [33]. Bge cells (~1.5 x 10^5^) in suspension were washed 3X with CBSS before incubation in CBSS (control), CBSS containing either 30 μg/mL LTP, 1 mM ZnCl_2_ or 30 μg/mL LTP + 1 mM ZnCl_2_ for 1 hr at 26°C. After treatment, cells were washed 3X with CBSS and centrifuged at 1000 rpm for 10 min. The final cell pellets were then re-suspended in 150 μL of CBSS containing 10 μM DCFH-DA, and distributed in three wells of a 96-well black-walled plate (BD Falcon). The oxidation of DCFH-DA to fluorescent 2’7’-dichlorofluorescein (DCF) was measured in triplicate at 10 min intervals for up to 60 min using a Bio-Tek Synergy fluorescence plate reader (Winooski, VT) with excitation and emission wavelengths of 485 ± 20 and 528 ± 20, respectively. Data analysis was conducted with Origin software (Microcal, Northhampton, MA, USA). Five independent replicates of each experiment were conducted, with the raw data presented as mean ± SEM, and ratios of means of treated groups to controls presented separately.

### Amplification and sequencing of H^+^ channel transcripts

For molecular analysis of H^+^ channels, the hydrogen voltage gated channel 1 (HVCN1) gene was identified in the nonredundant NCBI database, and sequence comparisons were conducted with PCR products from Bge cells and *B*. *glabrata* hemocytes. Schistosome-susceptible (NMRI) and resistant (BS-90) *B*. *glabrata* strains were maintained in laboratory colonies in 10-gallon aquaria at 26°C under 12:12 hr light/dark cycling. Hemolymph, containing hemocytes, was collected by headfoot retraction [34] and immediately transferred to Eppendorf tubes containing an equal volume of CBSS on ice. Hemocytes were then pelleted by centrifugation at 1000 RPM for 10 min and washed 3 times in CBSS. Bge cells, grown in a flask to ~80% confluency, were detached mechanically using a cell scrapper, transferred to a 15 mL conical tube and pelleted by centrifugation as described for hemocytes.

Total RNA was extracted from Bge cells and hemocytes of both *B*. *glabrata* strains using TRIzol reagent. Normalized concentrations of isolated total RNA samples were subjected to cDNA synthesis reactions using the GoScript^TM^ Reverse Transcription System (Promega Corp., Madison, WI). The cDNA was then used as the template for PCR using primers for the *B*. *glabrata* voltage-gated H^+^ channel 1-like gene (*Bg*HVCN1-like; Forward 5’-TGCTATGGGCTTAGCTTACTTC-3’; Reverse 5’-ATGTAGGGTCTTCAAACCATTCT-3’) that were designed using the predicted mRNA sequence for the gene with the National Center for Biotechnology Information (NCBI) database (Accession number XM_013231505). The expected amplicon size is ~362 bp, ~65% of the coding DNA sequence. As a positive control, primers for *B*. *glabrata* α–tubulin (Forward 5’ -GTGAGACTGGCTGTGGTAAA-3’; Reverse 5’ -GGGAAGTGAATCCTGGGATATG-3’) with Accession number XP_013094834.1 were used to amplify an expected product of ~643 bp. Gel electrophoresis of the PCR products was performed followed by Big Dye sequencing at the University of Wisconsin Biotechnology Center DNA Sequencing Facility (Madison, WI). The resulting nucleotide sequences were used in a search using BLASTn search against the non-redundant nucleotide NCBI database to confirm that the PCR amplified product encoded an HVCN1-like protein.

### Analysis and statistics

Patch clamp data were analyzed with Clampfit (Molecular Devices, Sunnyvale, CA) and Origin Pro (Microcal, Northhampton, MA). One-way RM-ANOVA and post-hoc statistical analyses were conducted in Origin Pro to assess significance. Results are presented as means ± SEM. The asterisks (*) represent p < 0.05 in all figures.

## Results

**Fig 1:**
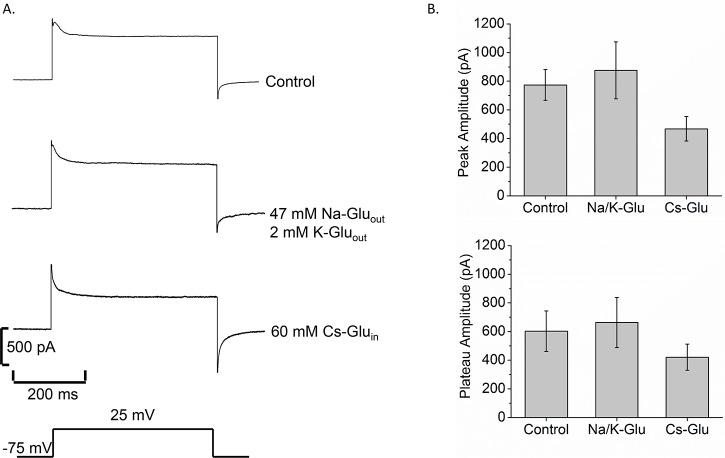
Bge cell membrane current is not carried by K^+^ or Cl^-^. A. Current traces in response to a voltage step from -75 mV to 25 mV for 500 msec for control solutions (top), 47 mM Na-Glu_out_/2 mM K-Glu_out_, with gluconate replacing Cl^-^ in the bathing solutions (second trace), and 60 mM Cs-Glu_in_ replacing KCl in the internal solution (third trace). The bottom pulse represents the voltage applied to the cell. B. Bar graphs show the mean peak current (top), and mean plateau current (bottom) for each group. N = 15 (control); N = 7 (Na/K Glu); N = 5 (Cs-Glu).

Whole cell patch clamp recordings were made from Bge cells to explore their membrane properties. Voltage steps from -75 to 25 mV for 500 msec induced an outward current that activated rapidly and then weakly inactivated in ~10–20 msec before stabilizing (Fig 1A, control trace, top). To identify the ions responsible for this current, we manipulated the composition of the recording solutions. When Cl^-^ was replaced by gluconate in the internal and bathing solutions, voltage steps induced currents similar to those seen with control solutions (Fig 1A, second trace from top). Further substitution of Cs^+^ for K^+^ in the internal solution reduced the current to roughly 68% of control currents (Fig 1A, third trace from top). The mean peak and plateau current amplitudes for these solutions are shown in Fig 1B. For gluconate and Cs^+^ substitution, current amplitudes were not significantly different from the control. Thus, Cl^-^ and K^+^ replacement experiments indicated that these are not major permeating ions. In addition, comparisons of the Nernst potentials (equilibrium potential for each ion based on internal and external concentrations) with reversal potentials in current-voltage relationships did not support channels selective for Na^+^ or Ca^2+^ (Supplemental S1 Fig). These results suggested that the major ions in our recording solutions do not permeate the membranes of Bge cells.

**Fig 2:**
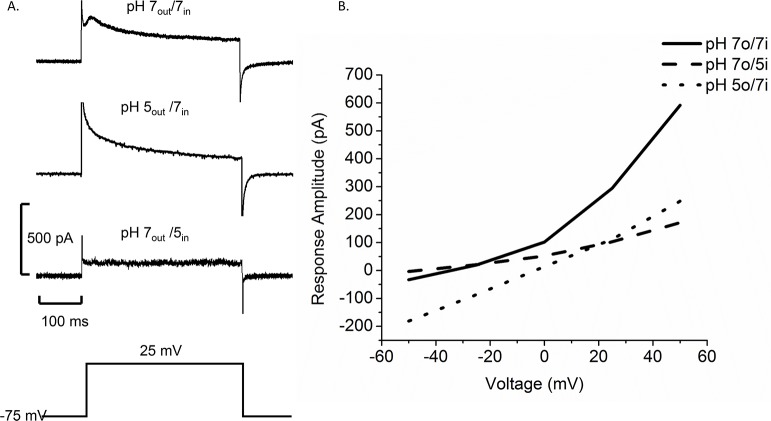
The pH gradient influences the voltage dependence of membrane current in Bge cells. A. Current traces show a control current with pH 7_out_/pH 7_in_ (top), pH 5_out_/pH 7_in_ (middle) and pH 7_out_/pH 5_in_ (bottom) (pulse from -75 mV to 25 mV indicated below). B. Plot of peak current versus voltage for steps varying from -50 mV to 50 mV. The solid curve represents control current in symmetrical pH (N = 4), the dotted curve represents pH 5_out_/pH 7_in_ (N = 5), and the dashed curve represents pH 7_out_ /pH 5_in_ (N = 5).

H^+^ channels play important roles in many types of immune cells [35], so we explored the possibility that H^+^ channels reside in the membranes of Bge cells. Subjecting Bge cells to pH gradients (by adjusting the pH of the pipette and bathing solutions–see Methods) [36] altered the current elicited by voltage steps and shifted the relationship between current and voltage (Fig 2). A gradient of two pH units (pH 5_in_/pH 7_out_) reduced the current amplitude at all voltages and shifted the reversal potential in the plot of peak current versus voltage in the negative direction by 17.5 mV (Fig 2B, dashed line). Reversing the pH gradient (pH 5_out_/pH 7_in_) shifted the peak current-voltage plot in the opposite direction with a positive shift in the reversal potential of 27.5 mV (Fig 2B, dotted line). Plots of plateau current versus voltage showed similar shifts (Supplemental S2 Fig). Table 1 presents the reversal potentials along with the Nernst potentials for H^+^. The shifts are in the direction of the H^+^ Nernst potential but smaller in magnitude because the H^+^ concentration is much lower relative to the concentrations of other ions in the solutions. Channels permeable to other ions generally result in H^+^ current reversal potential shifts that are less than the change in the H^+^ Nernst potential [37]. The effects of pH gradients on membrane currents are consistent with the presence of an H^+^ channel in Bge cell membranes.

**Table 1:** pH-dependence of reversal potentials in Bge cells.

	pH 7_in_/pH 7_out_	pH 5_in_ /pH 7_out_	pH 7_in_ /pH 5_out_
V_rev_ (mV)	-32.5±2.53 (n = 4)	-50.0±0.65 (n = 5)	-5±1.5 (n = 5)
E_H+_ (mV)	0	-116	116

Table 1. The mean voltages at which the current reverses are shown in the top row. The computed equilibrium potential for H^+^ (E_H+_) is shown in the second row.

**Fig 3:**
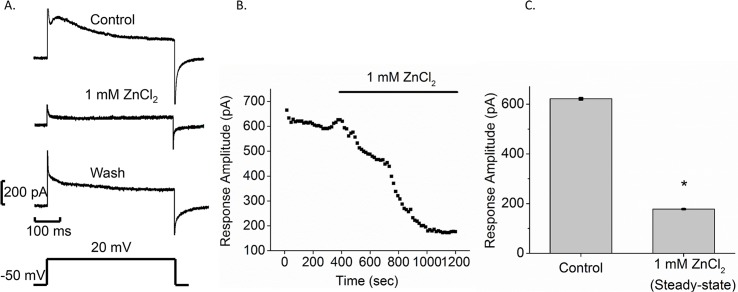
ZnCl_2_ blocks membrane current in Bge cells. A. Control response to a voltage step (from -50 mV to 20 mV indicated below) (top). One mM ZnCl_2_ was pressure applied onto Bge cells (middle). Wash response was measured10 min after post-ZnCl_2_ removal (lower). B. Time course plot is presented for perfusion of 1 mM ZnCl_2_. The bar labeled 1 mM ZnCl_2_ above represents the time of ZnCl_2_ application. C. Bar graphs show the mean current amplitude for control and in the presence of 1 mM ZnCl_2_ after full effect was reached (mean ± SEM; N = 4; *p < 0.05).

As an additional test for the presence of H^+^ channels we applied the H^+^ channel blocker Zn^2+^ [36, 38]. Pressure application of 1 mM ZnCl_2_ from a glass pipette onto a Bge cell significantly reduced both peak and plateau currents elicited by voltage steps from -50 to 20 mV (Fig 3A). This blockade was reversible, as demonstrated by current recovery after ZnCl_2_ removal (Fig 3A, wash trace). Time course plots in which ZnCl_2_ was perfused onto cells through the bathing medium showed a 3.5-fold reduction in current amplitude (Fig 3B and 3C), from 621 ± 4 pA to 177 ± 1 pA (N = 4), supporting the presence of H^+^ channels in Bge cell membranes. Although other actions of Zn^2+^ cannot be ruled out, the block of membrane current is consistent with the presence of H^+^ channels in Bge cells.

**Fig 4:**
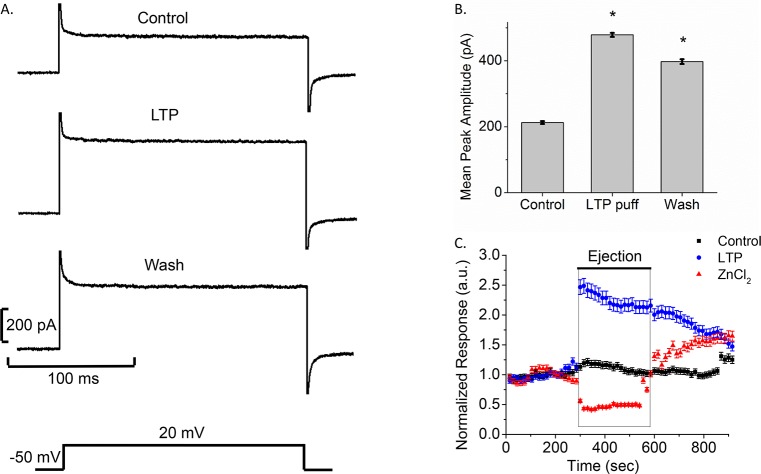
Larval transformation proteins (LTP) increase current amplitude in Bge cells. A. Traces for control (pre-LTP), during LTP pressure application (LTP), and washout (pulses from -50 mV to 20 mV as indicated below). B. Bar graphs show the mean peak amplitudes for each case (mean ± SEM; N = 5; *p < 0.05). C. LTP and ZnCl_2_ have opposite effects. Normalized time course is shown for control (black), ZnCl_2_ (red), and LTP (blue). The black bar above (labeled ejection) represents time of pressure application. Baseline responses were collected for 5 min, and the test solution applied for 5 min. For the control experiments, cells were puffed with CBSS. Responses were normalized by dividing by the average pre-puff baseline value. Note that puffing produces a more rapid effect than perfusion (compare with Fig 3). (mean ± SEM; N = 4-Control, 4-ZnCl_2_, 5-LTP).

**Fig 5:**
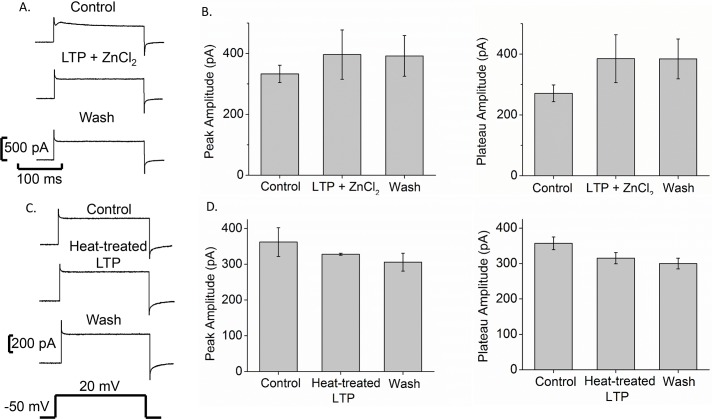
ZnCl_2_ counters the effect of LTP on H^+^ current in Bge cells. A. Simultaneous ZnCl_2_+LTP did not change peak current amplitude from control, although there was an ~100 pA difference in sustained current at the end of the pulse (from -50 mV to 20 mV indicated below). B. Bars show the mean peak and plateau current for each case (mean ± SEM, N = 10). C. Current traces from control (pre-puff), during heat-treated LTP (puff) and after washout show that heat-treated LTP does not significantly alter response amplitudes. D. Bar charts show mean peak and plateau current amplitudes before, during, and after application of heat-treated LTP (mean ± SEM, N = 3).

As larval schistosome proteins have been shown to modulate a variety of snail hemocyte immune functions [14, 15], we tested the effects of *S*. *mansoni* LTP on Bge cell membrane current. Pressure application of LTP onto Bge cells dramatically increased the peak and plateau currents evoked by steps from -50 mV to 20 mV (Fig 4A). LTP increased the current significantly by over 2-fold (478 ± 6 pA) compared to control (212 ± 4 pA), and this increase only partially reversed with a 17% decrease (397 ± 7 pA) following removal of LTP. Recovery was slow, and 5 min after LTP removal the current had decreased only slightly (Fig 4B and 4C). Plotting current versus time also illustrated the opposite effects of LTP and ZnCl_2_ on Bge cells (Fig 4C). This plot showed a >2-fold increase in current amplitude in the presence of LTP (Fig 4C blue circles) and a >2-fold reduction in the presence of ZnCl_2_ (Fig 4C, red triangles) compared to control (Fig 4C black squares). The reversal of block by ZnCl_2_ was rapid and essentially complete, while the reversal of enhancement by LTP was slow. Moreover, when heat-denatured LTP was pressure-applied onto Bge cells, we observed no significant change compared to control current amplitudes (Fig 5C and 5D), indicating that the action of LTP on H^+^ channels depends on heat-labile factors.

**Fig 6:**
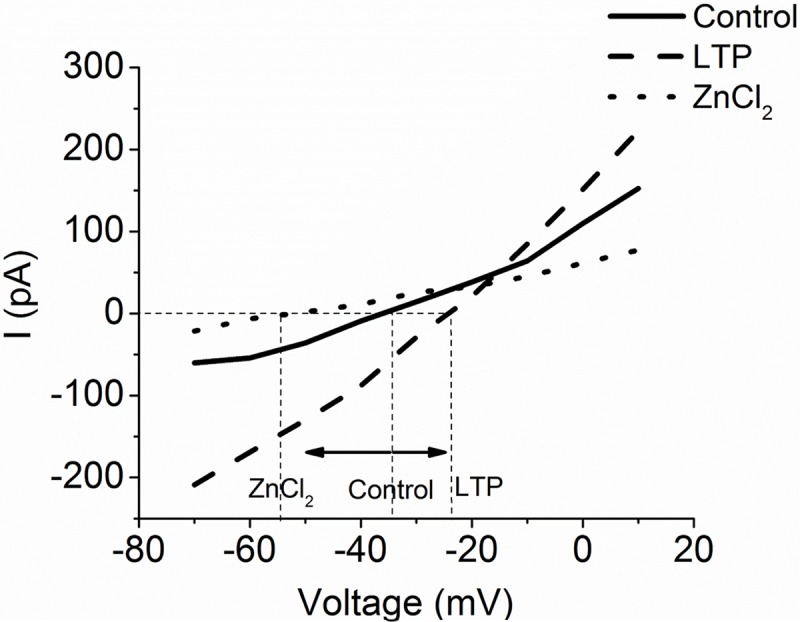
ZnCl_2_ (dotted line) caused a left-shift of the current-voltage plot from control (solid line), while LTP (dashed line) caused a right-shift. This supports blockage and opening of H^+^ channels, respectively. N = 4-Control, 6-ZnCl_2_, 5-LTP.

To determine whether LTP increased Bge cell membrane current by opening H^+^ channels, we applied LTP and ZnCl_2_ simultaneously, and observed no statistically significant change (Fig 5), indicating that ZnCl_2_ counters the effect of LTP. Finally, we noted that current-voltage curves shifted in the presence of LTP and ZnCl_2_; LTP caused a 9 mV right-shift from control, toward the H^+^ Nernst potential, while ZnCl_2_ caused a 23 mV left-shift, away from the H^+^ Nernst potential (Fig 6). These results are consistent with the blockade of H^+^ channels by ZnCl_2_ and enhancement of H^+^ channels by LTP.

**Fig 7:**
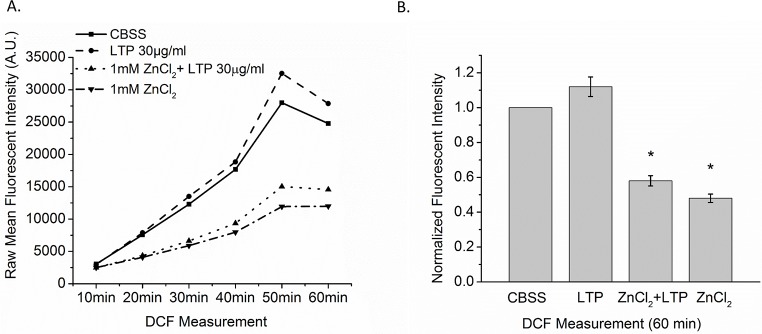
ROS production in Bge cells measured with DCFH-DA. Bge cells were exposed for 1 hr to CBSS only, LTP only, 1 mM ZnCl_2_ only or LTP + 1 mM ZnCl_2_. A. Raw DCFH-DA fluorescence was measured at 10 min intervals for 60 min and mean values plotted. ZnCl_2_ (downward triangle with dashed-dotted line) and LTP + ZnCl_2_ (upward triangle with dotted line) reduced fluorescence compared to CBSS control (square with solid line). LTP (circle with dashed line) had a weak, insignificant stimulatory effect. B. Bars show mean fluorescence with treatment normalized to constitutive control (CBSS) at 60 min. Bars represent means ± SEM. N = 5.

Because H^+^ channels contribute to ROS production in mammalian immune cells [35, 39], we measured the generation of ROS in Bge cells with the fluorescent probe 2’7’-dichlorofluorescein-diacetate (DCFH-DA). We observed a rapid and robust fluorescence increase that reflects constitutive ROS production. ZnCl_2_ and LTP + ZnCl_2_ inhibited this activity by ~50% compared to the untreated control (F_3, 16_ = 24.26, *p* < 0.05). These results demonstrate a linkage between H^+^ channels and the production of ROS in Bge cells. LTP alone produced a small apparent increase in ROS production, but this increase was not statistically significant. This suggests that at control level of H^+^ current in Bge cells, other factors limit ROS production (Fig 7A and 7B).

To identify putative H^+^ channel proteins expressed by Bge cells and *B*. *glabrata* hemocytes we searched the *B*. *glabrata* genome (https://www.vectorbase.org/organisms/biomphalaria-glabrata) using Blastp for homologues of human HVCN1 protein. The closest match was an HVCN1-like protein (*Bg*HVCN1-like, Accession number. XM_013231505) with 31% identity to human HVCN1. This sequence contained the motif RLWRVTR, which is consistent with the H^+^ channel consensus sequence RxWRxxR [36]. A segment of the predicted sequence was then used to design primers for polymerase chain reactions (PCR). Using cDNA from Bge cells and *B*. *glabrata* hemocytes (NMRI and BS-90 strains) as templates, PCR using the primers stated in the Methods section yielded amplicons of similar size with 99% sequence identity (E = 0.0) to the predicted *B*. *glabrata* HVCN1-like sequence (Supplemental S3 Fig). The amplified products encode 120 amino acid stretch of the 186 residues predicted for molluscan HVCN1-like protein. These results indicate that mRNA with the predicted sequence for a *Bg*HVCN1-like gene is present in both Bge cells and hemocytes.

## Discussion

This investigation revealed the presence of functional ion channels in Bge cell membranes. pH manipulations altered the voltage dependence of membrane currents in a manner consistent with a dominant H^+^ permeability. Since the H^+^ concentration was several orders of magnitude lower than the other ions in our solutions, even low permeabilities to other ions can make large contributions to the observed reversal potentials and move them away from the H^+^ Nernst potential. Thus, although currents did not reverse at the H^+^ Nernst potential, the shifts were in the appropriate direction and supported the hypothesis that H^+^ channels are the predominant ion permeability in the plasma membrane of Bge cells. We also found that the H^+^ channel blocker Zn^2+^ significantly reduced the current through Bge cell membranes, providing additional support for the presence of H^+^ channels. Finally, we identified and sequenced an HVCN1-like transcript expressed in both this snail cell line and *B*. *glabrata* hemocytes, suggesting a functional linkage between these cell types. Thus, three independent lines of evidence support the conclusion that Bge cells express functional H^+^ channels. With few exceptions [40], previous studies focusing on ion channels in molluscs almost exclusively have involved neuronal cell systems and/or emphasized Na^+^, K^+^, Ca^2+^ or Cl^-^ channel activities [41–43]. To our knowledge, this is the first report of a functional H^+^ channel in non-neuronal cells of freshwater gastropods.

Similar to the well documented association between H^+^ channels and ROS production in mammalian immunocytes [39], we also found that blockade of the H^+^ channel with Zn^2+^ significantly abrogated Bge cell ROS production, indicating a functional association between channel-mediated H^+^ flux across the membrane and the oxidative response. This finding is significant since the formation and release of several ROS, especially H_2_O_2_, and RNS are known to be involved in the killing of larval *S*. *mansoni* by *B*. *glabrata* hemocytes [9, 10]. It is possible that, as in mammalian immune cells [39, 44], changes in membrane potential associated with ROS production also require a compensatory activation of H^+^ channels to maintain pH balance in immunocyte-like molluscan cells. It is important to note that hemocytes from both resistant and susceptible strains of *B*. *glabrata* snails are capable of generating ROS [11, 32], but differ both qualitatively and quantitatively in their responses [11]. Since Bge cells were originally derived from a *S*. *mansoni*-susceptible Puerto Rican strain of *B*. *glabrata* [25], it is likely that hemocytes from a related susceptible strain (NMRI) also share both molecular and functional similarities to Bge cells. These shared characteristic have been well-documented in previous studies [26, 45, 46], supporting the use of this cell line as a hemocyte-like model, as well as a general model for *Biomphalaria-*schistosome interactions [29, 47]. Based on the presence and expression of the HVCN1-like gene in *B*. *glabrata* hemocytes, it is quite possible that voltage-gated H^+^ channels are also involved in regulating cellular ROS production as demonstrated in Bge cells.

Proteins released during the *S*. *mansoni* miracidium-to-sporocyst transformation (LTP) have been shown to modulate a variety of functions in both hemocytes and Bge cells [14, 24, 45]. Such a role is supported by our finding of an LTP-induced potentiation of H^+^ channel activity. Exposure to LTP elicited a rapid and sustained enhancement of Bge cell membrane current. Because the reversal potential moved toward the H^+^ Nernst potential, it is likely that LTP increased the current through H^+^ channels. This activity was heat-labile, suggesting that the channel-active LTP component(s) may be a protein(s) with irreversible or slowly reversing action. However, it remains unclear whether the regulation of Bge cell H^+^ channels by schistosome LTPs results from factors thought to play a role in host-parasite compatibility [48–50] or other, yet unidentified, larval factors. The H^+^ channel may play a role in co-evolutionary mechanisms, known to affect oxidant-antioxidant levels during parasite-host interaction [51]. Identifying the active components of LTP and determining whether this response reflects the action of a single or multiple species will require further investigation.

Despite the channel stimulating action of LTP, LTP treatment of Bge cells resulted in no statistically significant increase in ROS production. These results are consistent with previous findings that exposure of *B*. *glabrata* hemocytes to excretory-secretory products of larval *S*. *mansoni* exerted little effect on the production of ROS [52]. However, the question remains as to why LTP-stimulated H^+^ channel activation failed to enhance ROS production. Based on the H^+^ current data, it might be speculated that LTP binding to Bge cells is linked to the opening of H^+^ channels through receptor-mediated activation of a channel-associated signaling pathway, possibly through interactions with pathogen recognition receptors such as fibrinogen-related proteins, Toll-like receptors, or bacterial binding proteins that have been implicated in *B*. *glabrata* immunity [50, 53–55]. Mitogen-activated and extracellular-signal regulated protein kinases shown to function in molluscan immunity [12, 22] could also play a role in signaling to the H^+^ channel. A final possibility is that LTP may be acting directly on the channel protein itself to induce opening. The consequence of H^+^ channel modulation would be an alteration or disruption of H^+^ ion balance and intracellular pH, but without stimulating ROS production. This may, in turn, serve as a potent anti-immune mechanism used by sporocysts for countering host ROS-mediated effector responses. Thus, H^+^ channels, while serving an important role in maintaining pH balance within Bge cells and hemocytes, may also be manipulated by schistosome larvae to reduce their immune efficacy. Since Bge cells were originally derived from a *S*. *mansoni*-susceptible PR albino strain of *B*. *glabrata* [25], it is likely that hemocytes from a related susceptible strain (NMRI) also share sensitivity to H^+^ channel–reactive anti-immune proteins, thereby supporting a compatible snail-schistosome interaction.

In conclusion, Bge cells possess a functional H^+^ channel that is responsible for a dominant conductance of their plasma membrane. ROS production is dependent on H^+^ channels. Exposure of cells to heat-labile LTP stimulates channel opening and H^+^ flux, but has little if any effect on the generation of ROS. Although H^+^ channels have not been tested directly in *B*. *glabrata* hemocytes, PCR amplification and amplicon sequencing demonstrated the presence of HVCN1-like transcripts in both susceptible and resistant *B*. *glabrata* strains. Thus, the association of the Bge cell H^+^ channel activity with cellular ROS production and the channel’s response to schistosome LTP suggest a role in regulating larval schistosome-snail interactions. Future identification of the specific mechanism(s) tying together these activities should provide important insights into host-parasite compatibility in this system.

## Supporting information

S1 Fig Current-voltage plots with control solutions and K-gluconate solutions used in Fig 1. Current was measured for voltage steps varying from -50 mV to 50 mV. E_Ca_ was computed as +120 mV based on a free [Ca^2+^] of 200 nM computed from the EGTA and total [Ca^2+^] of the pipette solution. E_Na_ has a large positive value that could not be determined because the pipette solution had no added Na^+^. More positive voltage steps move the membrane toward the Nernst potentials for Na^+^ and Ca^2+^. The observed increases over the entire range with more positive voltage is not consistent with channels selective for Na^+^ or Ca^2+^.
(PDF)

S2 Fig The pH gradient influences the voltage dependence of membrane current in Bge cells. Current-voltage plot for voltage steps varying from -50 mV to 50 mV. Fig 2 plotted peak current and this figure plots plateau current in symmetrical pH (solid curve, N = 4), pH 5-out/7-in (dotted curve, N = 6), and pH 7-out/5-in (dashed curve, N = 5).
(PDF)

S3 Fig Gene sequence of HVCN1-like mRNA in Bge cells and *B*. *glabrata* hemocytes. PCR of cDNA derived from Bge cells and *B*. *glabrata* hemocytes of susceptible (NMRI) and resistant (BS90) snail strains revealed amplicons of predicted size (~362 bp) for *B*. *glabrata* HVCN1-like gene and the alpha tubulin (~643 bp) loading control (top). Multiple sequence alignment of HVCN1-like transcripts from Bge cells and hemocytes of *B*. *glabrata* (NMRI and BS90 strains) with the predicted sequence of *B*. *glabrata* HVCN1-like (PredBgHVCN1-like, Accession number XM_013231505) (bottom). The shaded regions show the minor differences in base pairs among the sequences.
(PDF)
