# Supplementary figures and images for: H+ channels in embryonic Biomphalaria glabrata cell membranes: Putative roles in snail host-schistosome interactions
Source: PLoS Negl Trop Dis. 2017 Mar 20;11(3):e0005467. doi: 10.1371/journal.pntd.0005467 (PMC5373640; doi:10.1371/journal.pntd.0005467)

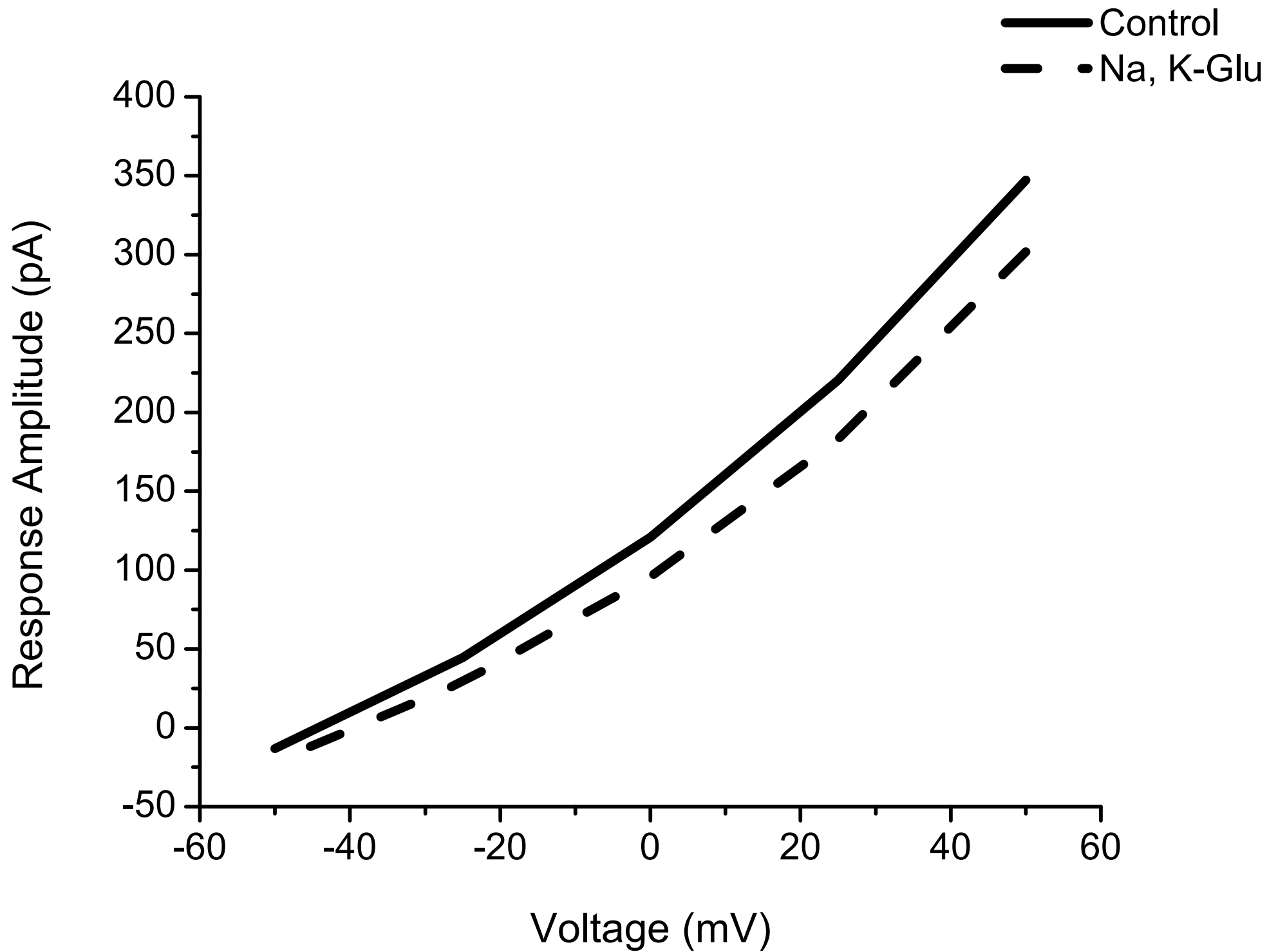

Supplement: S1 Fig — Current was measured for voltage steps varying from -50 mV to 50 mV. ECa was computed as +120 mV based on a free [Ca2+] of 200 nM computed from the EGTA and total [Ca2+] of the pipette solution. ENa has a large positive value that could not be determined because the pipette solution had no added Na+. More positive voltage steps move the membrane toward the Nernst potentials for Na+ and Ca2+. The observed increases over the entire range with more positive voltage is not consistent with channels selective for Na+ or Ca2+. (PDF) [file pntd.0005467.s001.pdf]

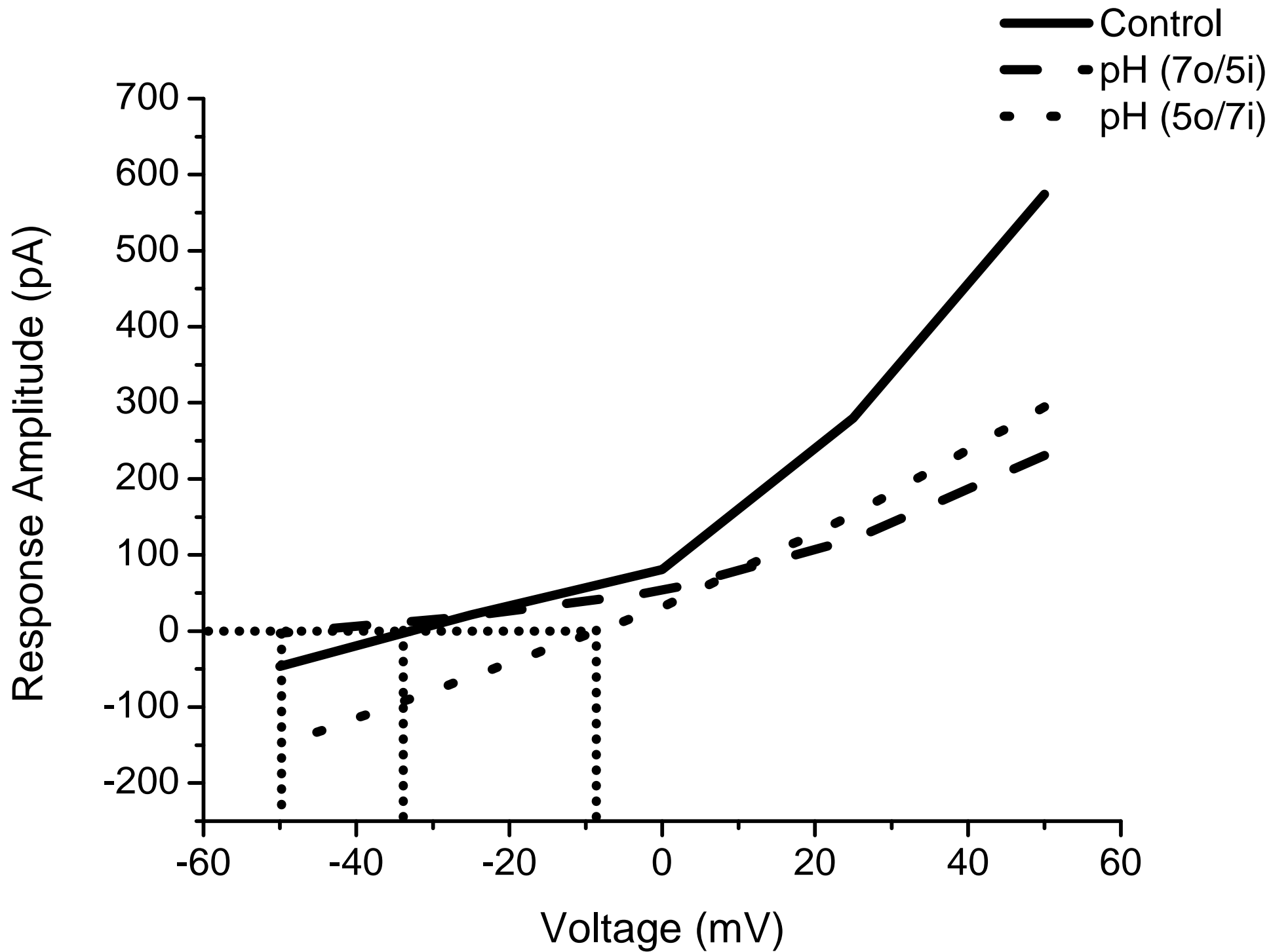

Supplement: S2 Fig — Current-voltage plot for voltage steps varying from -50 mV to 50 mV. Fig 2 plotted peak current and this figure plots plateau current in symmetrical pH (solid curve, N = 4), pH 5-out/7-in (dotted curve, N = 6), and pH 7-out/5-in (dashed curve, N = 5). (PDF) [file pntd.0005467.s002.pdf]
