# Supplementary material for: H+ channels in embryonic Biomphalaria glabrata cell membranes: Putative roles in snail host-schistosome interactions
Source: PLoS Negl Trop Dis. 2017 Mar 20;11(3):e0005467. doi: 10.1371/journal.pntd.0005467 (PMC5373640; doi:10.1371/journal.pntd.0005467)
Supplement: S3 Fig — PCR of cDNA derived from Bge cells and B. glabrata hemocytes of susceptible (NMRI) and resistant (BS90) snail strains revealed amplicons of predicted size (~362 bp) for B. glabrata HVCN1-like gene and the alpha tubulin (~643 bp) loading control (top). Multiple sequence alignment of HVCN1-like transcripts from Bge cells and hemocytes of B. glabrata (NMRI and BS90 strains) with the predicted sequence of B. glabrata HVCN1-like (PredBgHVCN1-like, Accession number XM_013231505) (bottom). The shaded regions show the minor differences in base pairs among the sequences. (PDF) [file pntd.0005467.s003.pdf]

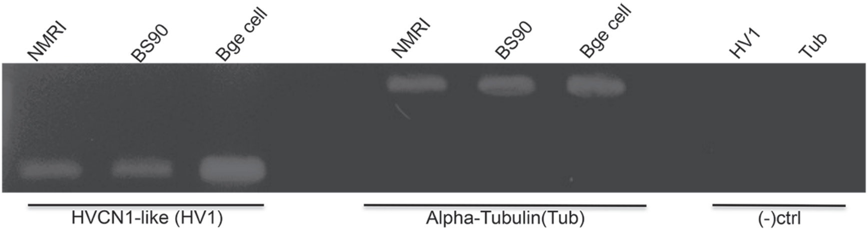


```
PredBGHVCN1-like   3  TGCTAT– GGG CTTAGCTT ACTTCACACATTTGTTAGAGGTCTTTGATGGCACAGTGATTA  61
Bge Cell           2  TGCTATG GGGGCTAGCTT ACTTCACACATTTGTTAGAGGTCTTTGATGGCACAGTGATTA  61
NMRI               2  TGCTATG GGGGCTAGCTT ACTTCACACATTTGTTAGAGGTCTTTGATGGCACAGTGATTA  61
BS90               2  TGCTATG GGGCTTAGCTT ACTTCACACA –TTGTTAGAGGTCTTTGATGGCACAGTGATTA  60

PredBGHVCN1-like  62  TTGTCTCCTTAATACTAGA CCTTGTCACATTGTCTCAGTCAGGGCTGGAA GTGATGGCAG  121
Bge Cell          62  TTGTCTCCTTAATACTAGA CCTTGTCACATTGTCTCAGTCAGGGCTGGAA GTGATGGCAG  121
NMRI              62  TTGTCTCCTTAATACTAGA CCTTGTCACATTGTCTCAGTCAGGGCTGGAA GTGATGGCAG  121
BS90              61  TTGTCTCCTTAATACTAGA CCCTGTCACATTGTCTCAGTCAGGGCTGGAA GTGATGGCAG  120

PredBGHVCN1-like 122  CTGATATGATCATTTTGTT CAGACTCTGG AGGGTGACACGTTT GTTTACAGGGTTCATCT  181
Bge Cell         122  CTGATATGATCATTTTGTT CAGACTCTGG AGGGTGACACGTTT GTTTACAGGGTTCATCT  181
NMRI             122  CTGATATGATCATTTTGTT CAGACTCTGG AGGGTGACACGTTT GTTTACAGGGTTCATCT  181
BS90             121  CTGATATGATCATTTTGTT CAGACTCTGG AGGGTGACACGTTT GTTTACAGGGTTCATCT  180

PredBGHVCN1-like 182  CCTCACTGAAGCGTCAGGCTGCAATCAAATCCATGGAGATAGAGGAGAAAAAAAAGGATT  241
Bge Cell         182  CCTCACTGAAGCGTCAGGCTGCAATCAAATCCATGGAGATAGAGGAGAAAAAAAAGGATT  241
NMRI             182  CCTCACTGAAGCGTCAGGCTGCAATCAAATCCATGGAGATAGAGGAGAAAAAAAAGGATT  241
BS90             181  CCTCACTGAAGCGTCAGGCTGCAATCAAATCCATGGAGATAGAGGAGAAAAAAAAGGATT  240

PredBGHVCN1-like 242  TAGAGAATCAGATTGAGC AATCCAATACAAATTGTAGAAGGC AAAAAGAGAGAATTGACT  301
Bge Cell         242  TAGAGAATCAGATTGAGC AATCCAATACAAATTGTAGAAGGC AAAAAGAGAGAATTGACT  301
NMRI             242  TAGAGAATCAGATTGAGC AATCCAATACAAATTGTAGAAGGC AAAAAGAGAGAATTGACT  301
BS90             241  TAGAGAATCAGATTGAGC AATCCAATACAAATTGTAGAAGGC AAAAAGAGAGAATTGACT  300

PredBGHVCN1-like 302  ATCTGACAAATCTTTTAAA AGATAACCATATTGTCTTTCAAGAATGGTTTGAA GACCC – T  360
Bge Cell         302  ATCTGACAAATCTTTTAAA AGATAACCATATTGTCTTTCAAGAATGGTTTGA – GACCC C T  360
NMRI             302  ATCTGACAAATCTTTTAAA AGATAACCATATTGTCTTTCAAGAATGGTTTGA – GACCC – T  359
BS90             301  ATCTGACAAATCTTTTAAA AGATAACCATATTGTCTTTCAAGAATGGTTTGA – GACCC – T  358

PredBGHVCN1-like 361  ACAT  364
Bge Cell         361  ACAT  364
NMRI             360  ACAT  363
BS90             359  ACAT  362
```
